# Association of serum total bilirubin levels with progressive renal decline and end-stage kidney disease: 10-year observational cohort study in Japanese patients with diabetes

**DOI:** 10.1371/journal.pone.0271179

**Published:** 2022-07-12

**Authors:** Erina Eto, Yasutaka Maeda, Noriyuki Sonoda, Naoki Nakashima, Kunihisa Kobayashi, Ryoichi Takayanagi, Yoshihiro Ogawa, Toyoshi Inoguchi

**Affiliations:** 1 Department of Medicine and Bioregulatory Science, Graduate School of Medical Sciences, Kyushu University, Fukuoka, Japan; 2 Medical Information Center, Kyushu University Hospital, Fukuoka, Japan; 3 Fukuoka City Health Promotion Support Center, Fukuoka City Medical Association, Fukuoka, Japan; The University of the West Indies, JAMAICA

## Abstract

**Objective:**

Previous reports have demonstrated the association of serum bilirubin levels with the progression of diabetic nephropathy. The objective of this study is to assess the association of basal bilirubin levels with progressive renal decline (PRD) and end-stage kidney disease (ESKD).

**Methods:**

A total of 298 patients with diabetes who visited Kyushu University Hospital (Japan) were recruited and followed up for 10 years. PRD was defined as a negative change in estimated glomerular filtration ratio (eGFR) >3.7%/year, 2.5th percentile. Logistic regression analysis was performed to evaluate the association of total bilirubin levels with PRD and its cut-off point was determined by receiver operating characteristic (ROC) analysis. Kaplan-Meier method and Cox hazard regression analysis were used to evaluate the predictive ability of its cut-off point for ESKD.

**Results:**

Logistic regression model showed that total bilirubin levels were significantly associated with PRD, and ROC analysis showed that its cut-off point was 0.5 mg/dL. Kaplan-Meier method showed that the percent of patients who reached two endpoints, composite endpoint (ESKD or doubling of creatinine level) or 30% eGFR decline, was significantly higher in the low bilirubin group than in the high bilirubin group (18.5% vs 11.0%, *P* = 0.045; 49.1% vs 42.1%, *P* = 0.045, respectively, log-rank test). Cox hazard regression models confirmed the independence of the predictive ability of its cut-off point.

**Conclusions:**

Serum total bilirubin levels were negatively associated with PRD in diabetic nephropathy and its cut-off point was 0.5 mg/dL. It may be clinically useful for identifying patients at high risk of ESKD.

## Introduction

Diabetic kidney disease is a leading cause of end-stage kidney disease (ESKD) worldwide. However, individual patients show a large variation in progression of kidney dysfunction [1–3]. It is therefore important to identify patients at high risk for developing ESKD. The estimated glomerular filtration rate (eGFR) is the most widely used parameter for the evaluation of changes in kidney function in clinical practice. Recent evidence has shown that rapid annual decline in eGFR is closely associated with subsequent progression to ESKD [4–6], and it is also associated with an increased risk of cardiovascular and all-cause mortality [6, 7]. Therefore, it is important to identify the predictive factors for such rapid decline in eGFR, so-called progressive renal decline (PRD).

In recent years, accumulating evidence has shown that oxidative stress may play an important role in the development of diabetic nephropathy [8–10]. However, most of intervention trials using various antioxidants failed to protect against it. Bilirubin is an important antioxidant enzyme, and it is a product of heme catabolism by heme oxygenase. It acts as a protective agent against oxidative stress damage [11]. We and other investigators have previously shown that serum total bilirubin levels are negatively associated with the development of diabetic complications including nephropathy [12–16]. We had shown for the first time that diabetic patients with Gilbert syndrome, a congenital hyperbilirubinemia, have a low prevalence of vascular complications including nephropathy [12]. One report has shown a significant graded inverse association between baseline serum total bilirubin levels and the progression of diabetic nephropathy in post hoc analysis in the Reduction of Endpoints in Non-Insulin Dependent Diabetes Mellitus with the Angiotensin II Antagonist Losartan (RENAAL) trial and in the Irbesartan Diabetic Nephropathy Trial (IDNT) [15]. A meta-analysis of 132,240 subjects from 27 studies also reported a significant negative association between serum total bilirubin levels and diabetic complications including diabetic nephropathy [16]. However, to our knowledge, there are no reports that showed the cut-off value of serum total bilirubin levels for identifying patients at high risk of PRD or ESKD.

The objective of this study was to evaluate the association of baseline serum total bilirubin levels with PRD so as to determine its cut-off value, and then confirm its effectiveness as a predictor for ESKD independent of various clinical and biological variables in a 10-year observational cohort study.

## Materials and methods

### Study population

In this study, patients with diabetes who were enrolled from April to June 2006 to the study we had reported before [12] were followed up at the Kyushu University Hospital until April 2016 for 10 years. A total of 298 patients were enrolled for the data analysis. These participants represent approximately 68% of the whole cohort of the patients enrolled from April to June 2006 (n = 429) after excluding the following; those who had incomplete laboratory data for analysis (n = 4), patients with eGFR more than 120 mL/min/1.73m^2^ (n = 16), those who suffered from hepatic diseases (n = 4) and other kidney diseases (n = 2) during the follow-up. In addition, patients with Gilbert syndrome were excluded in this study (n = 14). We excluded the participants who were followed up for less than one year (n = 91) (Participants recruitment and follow-up flow is shown in S1 Fig). All procedures were performed in accordance with the relevant guidelines and regulations. Informed consent was obtained in the form of opt-out on the web-site. Those who rejected were excluded. The study was approved by the ethics committees of the related institutes (Ethics Committee of Kyushu university hospital 27–191).

### Laboratory analyses

The total cholesterol, triglycerides, and high-density lipoprotein (HDL) cholesterol levels were measured using the COD-POD method Determiner TCII, the enzyme-mediated colorimetric method Determiner TGII, and homogenous assays MetaboLead HDL-C, respectively (Hitachi Kasei Co. Ltd., Tokyo, Japan). Low-density lipoprotein (LDL) cholesterol concentrations were calculated using the Friedewald equation. Hemoglobin A1c (HbA1c) values were determined using standard high-performance liquid chromatography methods. The HbA1c level was obtained using Japan Diabetes Society value at baseline. We calculated the value of the National Glycohemoglobin Standardization Program as Japan Diabetes Society values + 0.4 (%) [17]. Serum total bilirubin levels were measured using a commercially available kit (Wako, Osaka, Japan). Serum high-sensitive C-reactive protein (hs-CRP) was measured with a commercial kit (N-Latex CRPII; Dade Behring Marburg GmbH, Marburg, Germany). eGFR was calculated using the equation proposed by the Japanese Society of Nephrology [18].


$$eGFR\;\left(mL/min/1.73m^{2}\right)=194\times \;{Serum\;creatinine}^{-1.094}\times \;{Age}^{-0.287}\times \;0.739\;(if\;female)$$

Blood pressure was measured using a mercury sphygmomanometer with the participant sitting. Body mass index (BMI) was calculated as weight in kilograms divided by height squared in meters. The brachial-ankle pulse wave velocity was measured in the supine position after at least 5 minutes using the apparatus Form PWV/ABI (Omron Colin Medical Technology, Komaki, Japan).

### Baseline assessment

Hypertension was defined as systolic blood pressure ≥140 mmHg or diastolic blood pressure ≥ 90 mmHg, or the current use of any antihypertensive medication. Microalbuminuria was defined as a urinary albumin to creatinine ratio of 30–299 mg/g creatinine, and macroalbuminuria was defined as that of ≥300 mg/g creatinine. Retinopathy was assessed by a fundus examination by independent ophthalmologists, and graded as no diabetic retinopathy, single diabetic retinopathy, and preproliferative or proliferative retinopathy. Coronary artery disease was defined as a history of acute myocardial infarction, angina pectoris confirmed by clinically significant obstruction on coronary angiography, or revascularization with angioplasty or coronary artery bypass. Cerebrovascular disease was defined as a history of symptomatic stroke, confirmed by brain computed tomography or magnetic resonance imaging.

### Definition of PRD

PRD is defined as a negative eGFR change equal to or steeper than -3.7%/ year, which corresponds to the 2.5th percentile of the distribution of annual eGFR decline in the participants of this study. This criterion is in accord with those used in previous studies (ex. 3.3% per year [19], 4% per year [20, 21], 3 ml/min per 1.73 m^2^ per year [7]).

### Statistical analysis

A 2-sided *P* value of less than 0.05 was considered significant. Data are presented as mean ± standard deviation (SD) for variables with a normal distribution and as median (interquartile range) for variables with a non-normally distribution. The significance of differences was determined by the chi-squared test for categorical variables, and the unpaired t test or the Mann-Whitney test was used for continuous variables. We performed multivariate logistic regression analysis to assess the predictive value of baseline serum total bilirubin levels for PRD. The odds ratio (OR) and its corresponding 95% CIs for the risk of PRD was calculated per 0.1 mg/ dL increase in baseline serum total bilirubin levels. Then, we plotted the receiver operating characteristic (ROC) curve, and the optimal cut-off value of the baseline serum total bilirubin levels at baseline for PRD was obtained from the Youden index, the maximum (sensitivity + specificity– 1). An event-free survival curve for two endpoints, composite endpoint (ESKD or doubling of serum creatinine levels), or 30% reduction in eGFR, was estimated by the Kaplan-Meier method. The event-free survival rates were compared between patients with low serum total bilirubin levels (≤ cut-off value) and high serum total bilirubin levels (> cut-off value). Differences between groups were confirmed using log-rank test. Cox hazards regression models were also performed to assess the independency of baseline serum total bilirubin levels for the prediction of each endpoint. All analyses were conducted using JMP^®^ Pro 15.0 (SAS Institute Inc., Cary, NC, USA).

## Results

### Baseline characteristics of the study subjects

The patient characteristics are presented in Table 1. The median age was 63.5 years old. The mean body mass index (BMI) was 23.5 kg/m^2^. The median duration of follow-up was 9.0 years. During 10 years follow-up, 74 of 298 patients was classified into PRD.

**Table 1:** Characteristics of the study subjects at baseline.

Variables	n	
Gender (%)	298	
Male		163 (55)
Female		135 (45)
Age (years)	298	63.5 [56–70]
Body mass index (kg/ m^2^)	264	23.5±3.6
Cigarette smoking, current/ past (%)	280	45 (16)/ 82 (29)
Duration of follow-up (years)	298	9.0 [5.0–10.0]
Type 1 diabetes (%)	298	7 (2)
Hemoglobin A1c (mmol/ mol)	292	60 [52–73]
Hemoglobin A1c (%)	292	7.7 [7.0–8.9]
Duration of diabetes (years)	239	11 [5–18]
Systolic blood pressure (mmHg)	272	132±15.0
Diastolic blood pressure (mmHg)	272	74.7±10.6
Presence of hypertension (%)	285	165 (58)
Serum LDL cholesterol (mmol/ L)	276	3.05±0.86
Serum triglyceride (mmol/ L)	297	1.34 [0.91–2.13]
Serum uric acid (mg/ dL)	298	5.1 [4.0–6.0)
Serum total bilirubin (mg/ dL)	298	0.6 [0.5–0.8]
Serum creatinine (mg/dL)	298	0.75 [0.61–1.0]
Estimated glomerular filtration rate (ml/ min per 1.73 m^2^)	298	75.1±20.6
High-sensitivity CRP, (ng/ mL)	246	605 [267–1373]
ba-Pulse Wave Velocity (cm/ sec)	259	1742 [1494–2086]
Neuropathy (%)	288	138 (48)
Retinopathy (%)	292	122 (42)
None (%)		170 (58)
Simple (%)		53 (18)
Preproliferative or proliferative (%)		69 (24)
Nephropathy	297	
Normoalbuminuria (%)		183 (61)
Microalbuminuria (%)		64 (22)
Macroalbuminuria (%)		50 (17)
Ischemic heart disease (%)	296	37 (13)
Cerebrovascular disease (%)	296	32 (11)
Treatment of diabetes (%)	275	
Diet only		46 (17)
Oral hypoglycemic agents		154 (56)
Insulin		75 (27)
Renin-angiotensin system blockade use (%)	295	106 (36)

Abbreviations: HbA1c, hemoglobin A1c; LDL cholesterol, low density lipoprotein cholesterol; CRP, C-reactive protein; SI conversion factors: To convert bilirubin to μmol/ L, multiply by 17.104; high-sensitivity CRP to nmol/ L, multiply by 9.524. Data are presented as mean ± S.D. for variables with normal distribution and as median [interquartile range] for variables with non-normally distribution. Nominal data are presented as the total number of patients with percentages.

### Determination of cut-off value of serum total bilirubin levels

**Fig 1:**
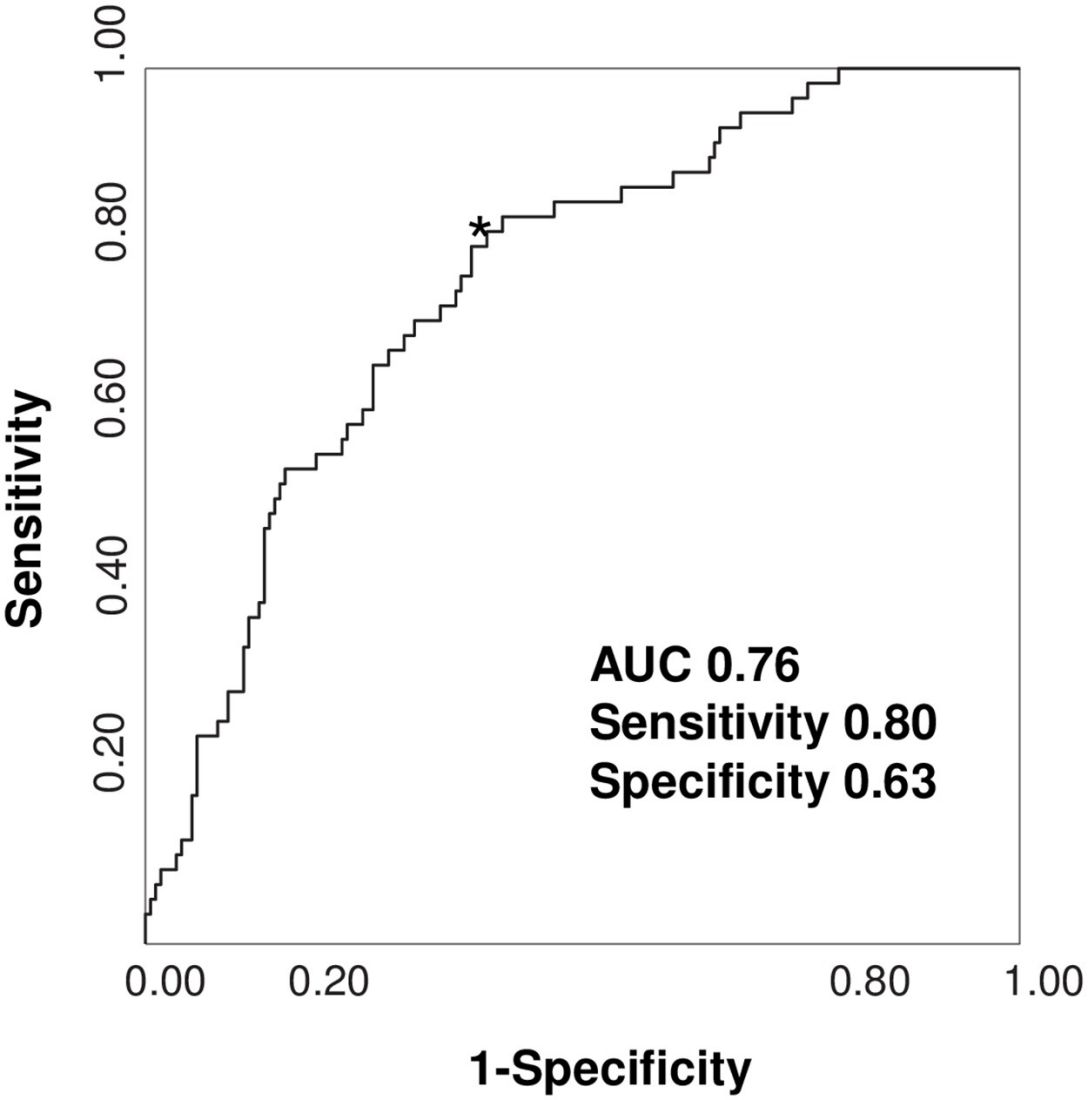
Determination of the cut-off value of baseline serum total bilirubin levels for predicting Progressive Renal Decline (PRD) by ROC curve. The area under the curve (AUC) of receiver operating characteristic (ROC) analysis for progressive renal decline was 0.76, with sensitivity 0.80 and specificity 0.63, and the cut-off value of baseline serum total bilirubin levels were 0.5 mg/dL.

The multivariate logistic regression analysis using baseline serum total bilirubin levels and conventional risk factors including age, gender, current smoker, body mass index (BMI), HbA1c, serum LDL-cholesterol, triglyceride levels, and the presence of hypertension was performed to assess the predictive ability of baseline serum total bilirubin levels for PRD. As shown in Table 2, the analysis showed that baseline serum total bilirubin levels, age, the presence of hypertension and serum triglyceride levels were significant predictors (OR 0.812, 95% CI 0.670–0.970, *P* = 0.026; OR 1.048, 95% CI 1.012–1.088, *P* = 0.012; OR 2.498, 95% CI 1.248–5.225, *P* = 0.012; OR 1.004, 95% CI 1.002–1.008, *P* = 0.005, respectively). The ROC analysis showed that its area under the curve (AUC) for PRD was 0.76 with sensitivity 0.80 and specificity 0.63, and the cut-off value of baseline serum total bilirubin levels were 0.5 mg/dL, as shown in Fig 1.

**Table 2:** Multivariate logistic regression model predicting progressive renal decline (PRD).

Variables	Univariate		Multivariate	
	OR (95%CI)	*P*	OR (95%CI)	*P*
Serum total bilirubin (/0.1 mg per dL)	0.876 [0.762–1.000]	0.0495	0.812 [0.670–0.970]	0.026
Gender (female/male)	1.035 [0.611–1.754]	0.898	0.900 [0.435–1.854]	0.771
Age	1.027 [1.000–1.054]	0.043	1.048 [1.012–1.088]	0.012
Body mass index	1.052 [0.974–1.136]	0.196	1.004 [0.910–1.106]	0.940
Current smoker	1.370 [0.682–2.753]	0.382	1.722 [0.667–4.318]	0.251
HbA1c	1.135 [0.971–1.327]	0.116	1.111 [0.910–1.355]	0.297
Presence of hypertension	2.516 [1.396–4.534]	0.001	2.498 [1.248–5.225]	0.012
Serum LDL cholesterol	1.000 [0.992–1.008]	0.946	1.004 [0.994–1.014]	0.459
Serum triglyceride	1.004 [1.002–1.007]	<0.001	1.004 [1.002–1.008]	0.005
n	298		228	

Abbreviations: HbA1c, hemoglobin A1c; LDL cholesterol, low density lipoprotein cholesterol. SI conversion factors: To convert bilirubin to μmol/ L, multiply by 17.104.

### The characteristics of patients stratified by optimal cut-off value

The characteristics of patients stratified by the optimal cut-off value (0.5 mg/dL) of baseline serum total bilirubin levels are shown in Table 3. Serum triglyceride levels, hs-CRP levels, and the presence of preproliferative or proliferative retinopathy, microalbuminuria, and macroalbuminuria and the rate of insulin therapy were significantly higher (*P* = 0.003, *P* = 0.004, *P* = 0.046, *P* = 0.033, *P*<0.001, and *P* = 0.007, respectively), and baseline eGFR and the rate of diet only therapy was significantly lower (*P* = 0.017, and *P* = 0.033, respectively) in the low bilirubin group (baseline serum total bilirubin levels ≤ 0.5 mg/ dL) than in the high bilirubin group (baseline serum total bilirubin levels > 0.5 mg/ dL).

**Table 3:** Comparison in variables between two groups divided by the cut-off value of baseline serum total bilirubin levels.

Variables	Serum total bilirubin levels≤0.5		Serum total bilirubin levels>0.5		*P*
	n		n		
Gender (%)	108		190		
Male		52 (48)		111 (58)	0.087^a^
Female		56 (52)		79 (42)	
Age (years)	108	64 [57–72]	190	63 (56–70)	0.450^b^
Body mass index (kg/ m^2^)	98	23.6±3.6	166	23.5±3.6	0.414^c^
Cigarette smoking, current/ past (%)	102	19 (19)/40 (39)	178	27 (15)/87 (49)	0.389/0.118^a^
Duration of follow up (years)	108	8.4 [4.7–10.0]	190	9.1 [5.2–10.0]	0.411^b^
Hemoglobin A1c (mmol/ mol)	106	60 [55–77]	186	60 [51–69]	0.179^b^
Hemoglobin A1c (%)	106	7.7 [7.2–9.2]	186	7.7 [6.9–8.5]	0.179^b^
Duration of diabetes (years)	91	11 [5–19]	148	10 [5–17]	0.411^b^
Systolic blood pressure (mmHg)	103	132±16.4	169	132±14.2	0.488^c^
Diastolic blood pressure (mmHg)	103	73.4±10.9	169	75.6±10.4	0.950^c^
Presence of hypertension (%)	104	65 (63)	181	100 (55)	0.233^a^
Serum LDL (mmol/ L)	99	2.97±0.79	177	3.10±0.90	0.902^c^
Serum triglyceride (mmol/ L)	107	1.72 [1.00–2.39]	190	1.28 (0.84–1.81)	0.003^b^
Serum uric acid (mg/ dL)	108	5.1 [4.0–6.0]	190	5.0 (3.9–6.1)	0.808^b^
Serum total bilirubin (mg/ dL)	108	0.5 [0.4–0.5]	190	0.7 (0.6–0.9)	<0.001^b^
Estimated glomerular filtration rate (ml/ min per 1.73 m^2^)	108	71.3±23.0	190	77.2±18.9	0.017^c^
High-sensitivity CRP, (ng/ mL)	91	687 [348–2420]	155	469(243–1130)	0.004^b^
ba-Pulse Wave Velocity (cm/ sec)	96	1844 [1509–2149]	163	1692 [1492–2042]	0.132^b^
Neuropathy (%)	102	52 (51)	186	86 (46)	0.441^a^
Retinopathy (%)	106		186		
None		57 (54)		113 (61)	0.225^a^
Simple		17 (16)		36 (19)	0.489^a^
Preproliferative or Proliferative		32 (30)		37 (20)	0.046^a^
Nephropathy					
Microalbuminuria (%)	86	18 (21)	134	46 (34)	0.033^a^
Macroalbuminuria (%)	108	32 (30)	189	18 (10)	<0.001^a^
Ischemic heart disease (%)	107	13 (12)	189	24 (13)	0.891^a^
Cerebrovascular disease (%)	107	15 (14)	189	17 (9)	0.181^a^
Treatment of diabetes (%)	104		171		
Diet only		11 (11)		35 (20)	0.033^a^
Oral hypoglycemic agents		55 (53)		99 (58)	0.417^a^
Insulin		38 (37)		37 (22)	0.007^a^
Renin-angiotensin system blockade use (%)	107	40 (37)	188	66 (35)	0.695^a^

Abbreviations: HbA1c, hemoglobin A1c; LDL, low density lipoprotein cholesterol; CRP, C-reactive protein; SI conversion factors: To convert bilirubin to μmol/ L, multiply by 17.104; high-sensitivity CRP to nmol/ L, multiply by 9.524. Data are presented as mean ± S.D. for variables with normal distribution and as median [interquartile range] for variables with non-normally distribution.

^a^ Calculated using the chi-squared test.

^b^ Calculated using the Mann-Whitney test.

^c^ Calculated using the unpaired t test.

### Predictive ability of cut-off value for renal endpoint by Kaplan-Meier method

**Fig 2:**
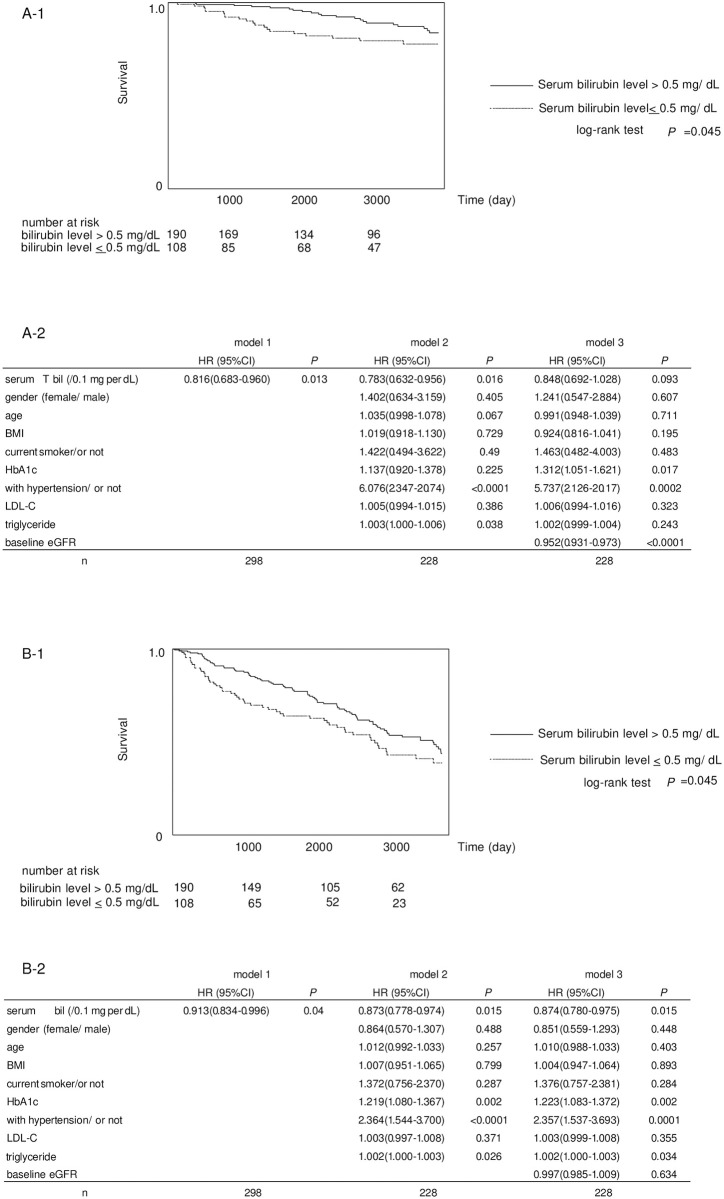
Comparison in renal endpoints between low bilirubin group (≤0.5 mg/dL) and high bilirubin group (>0.5 mg/dL) by Kaplan-Meier method and Cox hazard regression models showing the independence of baseline serum total bilirubin levels to predict renal endpoints. (a) The cumulative incidence of composite endpoint (end-stage kidney disease or doubling of serum creatinine) levels by Kaplan-Meier method are shown at upper panel. Cox hazards regression model was used to analyze the independence of baseline serum total bilirubin levels to predict the composite endpoint, adjusted for age, gender, BMI, current smoker, HbA1c, the presence of hypertension, LDL-cholesterol, triglyceride levels, and baseline eGFR (shown at lower panel). (b) The cumulative incidence of 30% reduction in estimated glomerular filtration rate by Kaplan-Meier method are shown at upper panel. Cox hazards regression model was used to analyze the independence of baseline serum total bilirubin levels to predict 30% reduction in estimated glomerular filtration rate, adjusted for age, gender, BMI, current smoker, HbA1c, the presence of hypertension, LDL-cholesterol, triglyceride levels, and baseline eGFR (shown at lower panel). T. bil, serum total bilirubin level; HR, hazard ratio; BMI, body mass index; HbA1c, hemoglobin A1c; LDL cholesterol, low density lipoprotein cholesterol, eGFR; estimated glomerular filtration rate. SI con-version factors: to convert bilirubin to μmol/L, multiply by 17.104.

Kaplan-Meier method showed that the percent of patients who reached the endpoint (ESKD or doubling of serum creatinine levels) were significantly higher in the low bilirubin group (20 of 108 [18.5%]) than in the high bilirubin group (21 of 190 [11.0%]) (*P* = 0.045, log-rank test) (Fig 2a). Similarly, the percent of patients who reached 30% eGFR decline were significantly higher in the low bilirubin group (53 of 108 [49.1%]) than in the high bilirubin group (80 of 190 [42.1%]) (*P* = 0.045, log-rank test) (Fig 2b).

### Predictive ability of cut-off value for renal endpoint by Cox hazard regression analysis

Finally, the Cox hazards regression model was used to analyze the independence of serum total bilirubin levels to predict renal endpoints, adjusted for age, gender, BMI, smoking status, HbA1c, the presence of hypertension, LDL-cholesterol, and triglyceride levels. Serum total bilirubin levels were significant independent predictors for ESKD (HR 0.783, 95% CI 0.632–0.956, *P* = 0.016, Fig 2a model 2). However, when baseline eGFR was added into the models, this association was attenuated and did not remain statistically significant (HR 0.848, 95%CI 0.692–1.028, *P* = 0.093, Fig 2a model 3). Nevertheless, for 30% eGFR decline, serum total bilirubin levels were significant predictors (HR 0.873, 95% CI 0.778–0.974, *P* = 0.015, Fig 2b model 2) and this association still remained statistically significant when eGFR was added into the model (HR 0.874, 95% CI 0.780–0.975, *P* = 0.015, Fig 2b model 3).

## Discussion

The present study showed here that baseline serum total bilirubin levels were independent predictors for progressive renal decline, so called PRD, and the cut-off value of serum total bilirubin levels were 0.5 mg/dL. In this long-term follow-up study, we confirmed that low serum total bilirubin levels (≤ 0.5 mg/dL) were significant predictors for composite endpoint, ESKD or doubling of serum creatinine levels. Recently, 30% reduction in eGFR have been proposed to be an alternative endpoint for ESKD [6, 22]. The present study also showed that low serum total bilirubin levels (≤ 0.5 mg/dL) were also significant predictors for the 30% reduction in eGFR. These findings indicated for the first time that the cut-off value of serum total bilirubin levels (0.5 mg/dL) may be effective in predicting ESKD in diabetic nephropathy.

Oxidative stress has emerged as an important pathogenic factor in the development of diabetic nephropathy. Various oxidative stress markers have been reported to be increased in kidneys from animals and patients with diabetes [8–10]. Supplementation of antioxidants such as vitamin E and lipoic acid, or overexpression of superoxide dismutase has been reported to attenuate renal damage in rodent models with diabetes [23–25], although there has been little evidence showing the beneficial effect of anti-oxidants in human. Bilirubin is a strong endogenous antioxidant [11]. The reno-protective properties of bilirubin are likely due to its potent antioxidant properties. Bilirubin has been shown to be more effective at protecting lipids from oxidation than water-soluble antioxidant glutathione [26], and almost 30 times more potent toward the prevention of LDL oxidation compared to a lipid-soluble vitamin E analog [27]. In addition, bilirubin was reported to inhibit NAD(P)H oxidase, which is a major source for reactive oxygen species (ROS) production in various tissues including vascular tissues and phagocytes [28]. We had shown for the first time that diabetic patients with Gilbert syndrome have a lower prevalence of vascular complications including nephropathy than those without it, in parallel with lower levels of 8-hydroxy-2’-deoxyguanosine (8-OHdG), oxidative stress marker, and high sensitivity-CRP [12]. Fukui et al. reported that low serum total bilirubin levels were correlated with microalbuminuria [13]. Riphagen et al. found inverse association between baseline serum total bilirubin levels and the doubling of serum creatinine levels and ESKD during a median follow-up of 2.5 years in a post hoc analysis of the result of RENAAL trial and IDNT [15]. A meta-analysis of 132,240 subjects from 27 studies also reported a significant negative association between serum total bilirubin levels and diabetic complications including diabetic nephropathy [16]. The present study showed that low serum total bilirubin levels at baseline were independent predictors for PRD and determined its cut-off value. In this long-term prospective study, the predictive ability of its cut-off value for ESKD was confirmed by Kaplan-Meier method. Furthermore, the independency of baseline serum total bilirubin levels for the prediction of renal outcome was shown with Cox proportional regression hazard model. Taken together, the present findings suggested that low serum total bilirubin levels at baseline and its cut-off value (0.5 mg/dL) may be clinically useful for identifying patients at high risk of PRD and subsequent ESKD in diabetic nephropathy. However, to our knowledge, there has been no previous report to evaluate the cut-off value and its validity. These should be confirmed in future prospective and large-scale studies.

In rodents, we reported that oral administration of biliverdin, a precursor of bilirubin, inhibited albuminuria and the progression of renal mesangial expansion in diabetes, as well as normalization of oxidative stress, and hereditary hyper-bilirubinemic Gunn j/j rats exhibited less albuminuria, oxidative stress markers and histological abnormalities in the kidneys after onset of diabetes induced by streptozotocin, comparing with diabetic normo-bilirubinemic Gunn J/+ rats [29]. These findings supported that bilirubin may inhibit the development of diabetic nephropathy via its antioxidant effects. In recent years, chronic inflammation has been considered to play an important role in multiple organ damage including nephropathy. Notably, recent studies have shown that bilirubin has anti-inflammatory properties [30]. This was consistent with the present finding that hs-CRP levels were significantly higher in the low bilirubin group (≤0.5 mg/dL) than in the high bilirubin group (>0.5 mg/dL). It is therefore very likely that ant-inflammatory effect as well as anti-oxidative effect may contribute to the protective effect of bilirubin on nephropathy. Although the decreased serum total bilirubin levels may be associated with the increased risk of cardiovascular events [31–33], unfortunately, we did not obtain the data of cardiovascular events in this study. A significant cause for the lack of association between serum total bilirubin levels and cardiovascular disease might be the small group size. Further studies should be done to evaluate whether the cut-off value of serum total bilirubin levels we showed here might be applicable as the predictor for increased risk of cardiovascular events.

Serum total bilirubin levels are highly genetic, but also influenced by many environmental factors, including pathological conditions. Low bilirubin have been reported to be associated with smoking, diabetes, metabolic syndrome, chronic kidney disease (CKD), aging-related disability [34–37]. In this study, Cox hazard regression models showed that the association of baseline bilirubin with renal endpoints remained significant even after the model was adjusted for age, gender, BMI, smoking status, HbA1c, the presence of hypertension, LDL-cholesterol and triglyceride. Especially, oxidative stress increases with advancing stage of CKD [38, 39]. In addition, several reports have shown that serum total bilirubin levels are positively associated with eGFR levels in CKD [36, 40]. The present study showed that when baseline eGFR levels was added into these models, its association with composite endpoint was attenuated and did not remain statistically significant (HR 0.848, 95%CI 0.692–1.028, *P* = 0.093), nevertheless, its association with 30% eGFR decline still remained statistically significant. This finding suggested that baseline low serum total bilirubin levels may be significant independent predictors for ESKD, although its association might be in part mediated by baseline low eGFR levels. Bilirubin functions as an antioxidant in vivo by reacting with ROS and being consumed, and thus its serum levels can be decreased in increased oxidative stress condition. It is therefore possible that low serum total bilirubin levels may be in part results of progressive kidney damages which are in increased oxidative conditions. Taken together, it is most likely that low bilirubin and the progression of nephropathy may form a vicious cycle.

The strength of this study was long-term follow-up (median 9.0 years). The most important limitation of this study was its observational study design. Therefore, no information was available on whether therapeutic interventions such as glucose, hypertension, lipids or lifestyle management may affect renal endpoints or serum total bilirubin levels. Second, we measured only total bilirubin levels, but not indirect bilirubin levels. Since indirect bilirubin can permeate the cell membrane, elevated levels of serum indirect bilirubin may inhibit oxidative stress more efficiently in diabetic micro- and macro-vascular tissues, via both its inhibitory effect on NAD(P)H oxidase and its radical scavenging effect inside the cells. These possibilities should be clarified in future studies. Third, this study was single center study at the university hospital and sample size was small. Especially, the cut-off value of serum total bilirubin levels shown in this study should be confirmed in large-scale, multi-center studies in the future.

## Conclusions

Low serum total bilirubin levels were significantly associated with PRD in diabetic nephropathy, and the cut-off value of serum total bilirubin levels were 0.5 mg/dL. This cut-off value was effective in predicting composite endpoint (ESKD or doubling of serum creatinine levels) and 30% reduction in eGFR. In addition, bilirubin measurement is inexpensive, performed routinely, and accessible to most hospitals or clinics. Low serum bilirubin level and its cut-off value may be clinically useful for identifying patients at high risk of ESKD in diabetic nephropathy. The prospective studies are needed to confirm this.

## Supporting information

S1 Fig Participants recruitment and follow-up flow. (PDF)

S1 Dataset (PDF)

S1 Checklist STROBE statement—Checklist of items that should be included in reports of observational studies. (DOCX)
