# Supplementary figures and images for: Association of serum total bilirubin levels with progressive renal decline and end-stage kidney disease: 10-year observational cohort study in Japanese patients with diabetes
Source: PLoS One. 2022 Jul 12;17(7):e0271179. doi: 10.1371/journal.pone.0271179 (PMC9275719; doi:10.1371/journal.pone.0271179)

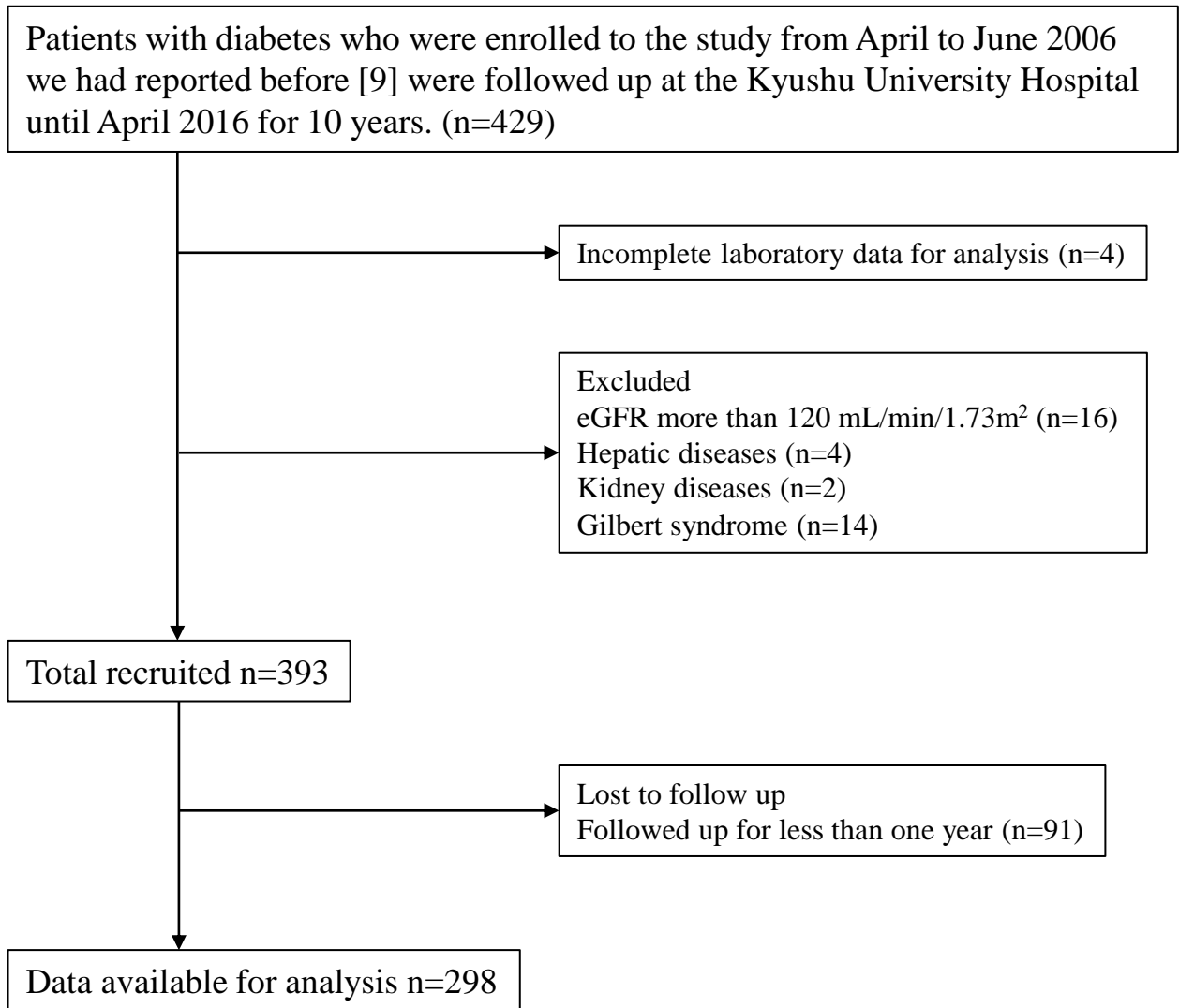

These participants represent approximately 68 % of the whole cohort.

Supplement: S1 Fig — (PDF) [file pone.0271179.s001.pdf]
