# Supplementary material for: Association of serum total bilirubin levels with progressive renal decline and end-stage kidney disease: 10-year observational cohort study in Japanese patients with diabetes
Source: PLoS One. 2022 Jul 12;17(7):e0271179. doi: 10.1371/journal.pone.0271179 (PMC9275719; doi:10.1371/journal.pone.0271179)
Supplement: S1 Dataset — (PDF) [file pone.0271179.s002.pdf]

ID	T B	Gender F	age	duration	BMI	sBP	dBP	HbA1c	LDL-C	TC	TG	HDL-C	retinopath	Proliferati	Simple	proteinuri	macro	micro	neuropath	IHD	CVD	rtPWV	ltPWV	hypertens	RA	diet only	OHA	insulin	hs-CRP	8-OHdG(	current	past	HD	baselinee	Cre
1	0.3	0	50	10	24.21875	96	49	10.9	108.2	174	69	52	0	0	0	0	0		1	0	0	1156	1189	0	0	0	1	0	158	8.650953	0	0	0	111.7448	0.57
2	1.1	1	55	5	19.3792	118	84	7.8	96.6	179	127	57	0	0	0	0	0		0	0	0	1316	1331	0	0	0	1	0			0	1	0	71.52923	0.9
3	0.3	0	66	21	27.34111	170	70	9.3	154.8	221	101	46	1	1	0	1	1	0	1	0	1	2243	2493	1	1	0	0	1	487	10.29114	0	0	0	60.00967	1.42
4	0.7	1	74	26		153	85	7.8		176	170		0	0	0	0	0	1	1	0	0	2483	2385	1	0				630	15.60274				72.0032	0.74
5	0.8	1	75	24	21.45357	135	60	6.4	96.2	177	104	60	1	1	0	0	0	1	1	1	1			1	1	0	0	1	407	16.33017	0	1	0	56.81124	0.63
6	1	1	77		23.38435	132	76	5.7	98.8	168	41	61	0	0	0	0	0		1	0	0	1529	1647	0	0	1	0	0			0	1		60.86767	0.81
7	1	1	67		21.19274	130	74	7.2	119.2	220	219	57	1	0	1	0	0	1	1	0	0	1692	1690	0	0	0	1	0	321	16.09599	0	1		77.24981	0.86
8	0.6	1	61	18	21.875	132	68	9.5	155.2	233	104	57	0	0	0	0	0		0	0	0	1593	1606	1	1	0	1	0	378	18.0545	1	1	0	89.47695	0.77
9	0.5	0	63	16	18.88889	138	70		119	179	85	43	1	0	1	0	0	1	0	0	0	1840	1812	0	0	0	1	0	183	10.69334	0	0	0	111.7649	0.43
10	0.6	0	65	20	21.28743	126	66	8.4	158.2	251	149	63	1	0	1	0	0	0	1	0	0			0	0	0	1	0	443	10.23162	0	0		78.51449	0.58
11	0.9	1	75		23.98687			5.8	95.4	173	198	38	0	0	0	0	0	0	0	0	0	2489	2698	1	1	0	1	0	819	9.276582	0	1	0	58.09395	0.82
12	0.5	1	71	9	22.94213	160	96	7.2	80.2	168	149	58				1	1	0	1	0	1	2984	2728	1	1	1	0	0	664	10.38858	1	1	0	56.0145	1.33
13	0.8	1	69					7.3	107.6	188	147	51	1	1	0	0	0	0	1	0	0			1	1				1680	11.68854	1	1	0	82.43854	0.68
14	0.3	1	58	29	21.41094	140	85	8.3	188	280	235	45	1	1	0	1	1	0	1	0	0	2134	2155	1	1	0	0	1	1250	4.521477	0	1	1	17.37491	5.15
15	0.7	1	74	20	18.06617	152	74	7.5	110.6	170	87	42	0	0	0	1	0	1	1	0	0	4189	3756	1	1	0	0	1	373	8.380196	0	1	0	69.16093	1.53
16	1	1	78	30	20.32444	126	62	7.1	152.4	240	48	78	1	0	1	0	0	1	1	0	0	2102	1913	1	1	0	1	0	313	5.169072	0	1		49.08243	1.23
17	0.4	0	74	5	19.43635	152	88	7	146.4	220	118	50	0	0	0	0	0	0	0	0	0	2248	2252	1	0	0	1	0	199	15.12627	0	0	0	80.17148	0.53
18	0.3	0	61	13	21.35931	132	62	7	106.4	201	68	81	0	0	0	0	0	0				1483	1546	0	0	0	1	0	13000	11.56565	0	0	0	90.10499	0.54
19	0.7	0	59	32	22.61719	130	76	9.2	121.2	218	69	83	1	1	0	0	0	1	0	0	0	1689	1654	1	1	0	0	1	207	15.70248	0	0	0	75.04731	0.64
20	0.7	1	70	16	22.9854	152	80	6.9	128.6	222	122	69	0	0	0	0	0	0	0	0	0	1762	1631	1	0	0	1	0	365	18.6317	0	1	0	68.46589	0.95
21	0.8	0	53	4	19.42798	132	84	6.5	157	263	170	72	1	1	0	1	1	0	1	0	0	1629	1695	1	1	0	1	0	702	11.19481	0	0		97.92776	0.73
22	0.6	1	56	10	21.12628	136	86	9.1	147.4	227	148	50	0	0	0	0	0		0	0	1	1757	1641	1	1	0	1	0	1870	10.94323	0	1	0	107.7422	0.67
23	1.2	0	72					8.6	109.2	164	109	33	0	0	0	1	1	0	0	0	0			1	1						0	0		57.55389	0.79
24	0.5	0	58	13	20.92747	110	76	7.6	73.4	190	328	51	0	0	0	0	0	0	0	0	0	1396	1484	0	0	0	0	1	555	13.61822	0	0	0	105.5461	0.51
25	0.7	1	70	8				7.4		207	75		0	0	0	0	0	0		0	0			1	0	0	1	0	485	13.49073	0	1		91.81875	0.62
26	1.1	0	75	10	26.47918	136	68	8.6	170.8	234	66	50	0	0	0	0	0		0	0	0	2221	2276	0	0	0	1	0			0	0		75.94303	0.5
27	0.5	0	74	4	21.77778	126	66	8.1	90	183	280	37	0	0	0	0	0	0	0	0	0	2187	2183	1	0	0	1	0	655	12.57602	0	0		62.08001	0.58
28	0.6	0	52	4	27.7671	122	88	9.2	120.6	238	342	49	0	0	0	0	0	0	0	0	0	1431	1432	0		0	1	0	793	8.207934	0	0	0	96.35437	0.51
29	0.5	1	49	22	22.92145	116	46	9.1	105	193	155	57	0	0	0	0	0	0	1	1	0	1269	1262	1	1	0	0	1	153	9.057802	0	1		77.55261	0.82
30	0.6	0	64	5	19.89912	134	82	6.6	131.4	226	278	39	1	0	1	0	0	1	1	0	0	2085	1925	0	0				15700	15.92304	0	0	0	81.95111	0.65
31	0.5	0	66	5	25.77778	122	74	9.4	157	219	95	43	1	0	1	0	0	0	1	0	0	1581	1563	1	0	0	1	0	7740	12.10971	0	0	0	108.4483	0.53
32	0.8	1	77	6	23.61275	130	80	7.5	69.2	172	294	44	1	0	1	0	0	1	0	0	0	2265	2338	1	1	0	1	0	938	18.77702	0	0	0	42.56878	1.15
33	0.6	1	72	17	23.24341	134	84	6.3	107.8	176	116	45	1	0	1	0	0	1	0	0	1	1681	1656	0	0	0	1	0	1440	7.970774	0	1	0	82.69337	0.59
34	0.7	1	52	19	30.83653	122	70	7.3	185.8	244	111	36	0	0	0	1	0	1	1	0	0	1441	1497	1	1	0	1	0	129	14.4524	1	1		80.78016	0.59
35	0.6	1	71	24	19.66904	144	68	6.7	81.8	155	51	63	1	0	1	0	0	1	0	0	0	1996	2034	1	0	0	0	1	257	10.36379	0	1	0	80.79984	0.74
36	0.7	1	79	15	24.03461	134	86	7.5	121.2	196	204	34	0	0	0	1	0	1	0	0	0	2183	2101	0	0	1	0	0	1730	6.058446	0	1	0	39.12421	2.1
37	0.8	0	71	23	24.06405	125	74	8.5	142.6	225	92	64	1	1	0	1	0	1	1	0	0	1823	1883	1	0	0	0	1	774	10.55607	0	0		114.9427	0.39
38	0.4	0	70	30	28.76397	136	72	12.7	115.2	206	84	74	1	1	0	0	0	0	1	1	1	2030	1881	1	1	0	0	1	273	5.245902	0	0	0	71.74968	0.84
39	0.6	1	55		30.96886					304	381		1	0	1	0	0		0	0	0	1386	1384		0						0	0	0	80.60577	0.81
40	0.6	1	31	3	31.17378	126	76	4.8	87.8	205	356	46	0	0	0	0	0		0	0	0	1212	1263	1	0	1	0	0	3280	11.26761	0	1	0	76.99595	0.85
41	0.8	0	68	8	19.39619	110	68	6.2	84	154	80	54	0	0	0	0	0	0	0	0	0	1768	1721	0	0	0	1	0	386	6.672328	0	0	0	75.43813	0.57
42	0.6	0	53		19.80537			14.1	97.2	188	79	75	0	0	0	1	1	0	1	1	0	1369	1403	1	1						0	0	0	92.47727	0.51
43	0.5	0	72	37	22.04916	112	54	9	79.2	138	94	40	1	0	1	1	1	0	1	1	0	2086	2182	1	1	1	0	0			0	0		31.14424	1.53
44	0.4	1	74	18	23.52941	130	82	6.2	92	186	320	30	0	0	0	1	1	0	0	0	0	2330	2310	1	1	1	0	0	569	10.01722	0	1		15.80149	4.15
45	0.8	0	82			183	106	8.8	88.4	203	428	29	1	1		0	0	1	1	0	0	3188	3193	1	1	0	1	0	781	19.23077	0	0	0	53.87171	0.72
46	0.7	0	57		27.44905			7.7		237	145		0	0	0	0	0		0	0	1	2122	2139	1	1						1	1	0	55.27125	0.82
47	1.2	0	71	5	22.22222	116	52	7.4	140	238	65	85	0	0	0	0	0	1	0	0	0	2293	2201	0	0				12600	15.17939	0	0	0	67.57839	0.54
48	0.5	1	65	5	24.4646	136	66	7.3	118.6	201	242	34	0	0	0	0	0	0	0	0	0	1523	1769	0	0	0	1	0	1470	12.57184	0	1	0	97.0548	0.64
49	0.3	1	76	37	24.5632	106	64	6.9	100.2	163	69	49	0	0	0	1	1	0	0	0	0	1618	1591	1	1	0	1	0	228	6.85258				40.19112	1.38
50	0.9	0	65	8	25.37778	124	70	6.1	121.4	210	83	72	0	0	0	0	0	0	0	0	0	1895	1916	1	1	1	0	0	139	18.15353	0	0		84.89874	0.61
51	0.6	0	66	50	22.36744	114	70	7.6	137.6	216	92	60	0	0	0	0	0	0	1	1	0	2059	2164	0	0	0	0	1	1070	11.31008	0	0	0	69.00965	0.64
52	0.5	0	75	10	24.06405	120	78	6.1	88.4	192	113	81	0	0	0	1	0		0	1	0	1929	1797	1	0	0	1	0	1110	9.682705	0	0		70.05305	0.65
53	0.8	1	73	31	26.44628	144	78	6.5	107.6	193	57	74	1	0	1	0	0	0	1	0	0	2378	2569	1	0	0	1	0	1010	26.81871	1	1		87.08299	0.64
54	0.6	1	73	9	26.23358	112	76	7.5	138	197	100	39	1	0	1	0	0	0	1	0	0	1719	1729	0	0	0	1	0	1530	4.547795	0	1	0	75.37151	0.8
55	0.8	1	65	4	22.79916	140	82	6.7	141.8	218	131	50	0	0	0	0	0	0	0	0	0	1781	1670	0	0	0			835	9.667396		0		79.04702	0.85
56	0.8	1	60	7	22.32143	120	78	7.1	120.6	204	62	71	0	0	0	0	0		0	0	0	1462	1466	0	0	1	0	0			0	0	0	99.31018	0.76
57	0.6	0	71	44	21.70957	122	56	9.8	84	177	295	34	1	1	0	0	0	0	1	1	1	2510	3173	1	1	0	0	1	1360	14.4889	0	0	0	75.64608	0.69
58	1	1	70	15	17.36044	132	62	6.1	89.4	252	93	144				1	1	0	1	0	1	2135	1924	1	1	1	0	0			0	1	0	84.6684	0.85
59	0.3	0	73	16	23.96822	130	62	7.4	105.2	183	189	40	0	0	0	1	1	0	0	0	0	2544	2272	0	0	0	1	0	17400	22.22222				42.93143	0.85
60	0.7	1	72	6	22.70454	144	62	5.9	78	179	80	85	0	0	0	0	0	0	0	0	0			1	0	0			171	15.11944	0	0	0	71.59203	0.74
61	0.3	0	70	28	29.05329	126	86	7.7	121.2	193	139	44	1	1	0	1	1	0	1	1	1			1	1	0	0	1	7940		0	0	0	25.50202	1.53
62	0.8	1	75	20	19.63659	126	90	9.9	136.4	257	298	61	1	0	1	0	0	0	0	1	0	2529	2713	1	1	0	0	1	14400	20.13943	0	0	0	87.53657	0.56
63	0.9	0	70	18	20.82466	126	78	7.1	107.8	207	41	91	1	0	1	0	0	0	0	0	0	1978	1896	1	1	0	1	0	388	14.23732	0	0	0	80.80439	0.58
64	0.5	0	83	23	24.23823	132	72	6.5	142	240	215	55	1	1	0	1	1	0	1	0	0	3381	1667	1	1	0	0	1	333	8.745411	0	0	1	25.88373	5.36
65	1.2	0	59	9	29.01745	126	90	6.8	128.2	200	139	44	0	0	0	0	0	0	0	0	0	1494	1579	1	0	0	1	0	2470	11.44297	0	0	0	85.55686	0.53
66	0.4	1	64	20	20.44359	128	78	7.1	95.8	248	586	35	1	0	1	1	1	0	1	0	1	1965	1339	0	0	0	0	1	1500	11.48518	1	1	1	79.50624	1.31
67	0.6	1	44	2	23.8806	102	68	6.3	121.6	181	142	31	0	0	0	0	0	0	0	0	0			0	0	0	1	0			1	1		95.24763	0.84
68	0.5	0	74	19				7.2	142	224	155	51	1	1	0	1	1	0	1	1	1	1865	2166	1	1	0	1	0			0	0		32.57017	2.03
69	0.6	1	57			120	66	10.1	97.6	166	157	37	1	0	1	0	0		1	1	1	1109	1352	1	1	0	0	1			1	1		80.121	0.7
70	0.5	1	76			116	60	8.8		198	59		0	0	0	0	0	0	1	1	0	1446	1437	0	0	0	0	1	178	12.40528	0	1		94.43473	0.58
71	0.7	1	62	20	27.34375	132	82	7.4	153.8	240	146	57	1	0	1	0	0	0	1	0	0	1659	1682	0	0	0	1	0	480	13.58305	0	0		87.66941	0.69
72	0.5	1	81	19	23.73321	140	72	6.9	75	143	55	57	0	0	0	0	0	0	0	0	0	2755	2763	1	1	0	1	0	640	22.05107	0	0	0	53.2136	1.19
73	0.7	0	66	19	22.59861	150	83	7.9	138.2	225	119	63	0	0	0	0	0		1	0	0	2788	2817	1	1	0	1	0	252	10.69736	0	0		54.98657	0.92
74	0.9	1	71	9	27.77671	136	76	10.1	219.4	310	113	68	0	0	0	0	0	1	1	0	1	1716	1799	1	1	0	0	1	1830	15.02827	1	1		98.63584	0.62
75	1	1	59	5	20.82094	114	68	5.7	171.4	269	213	55	0	0	0	0	0	0	0	0	0	1304	1252	0	0	0	1	0	255	15.04308	0	1		86.22743	0.65
76	0.6	1	61	18	18.10161	110	74	7.9	142.4	225	223	38	1	1	0	1	1	0	1	0	0	1706	1621	0	0	0	0	1	1200	10.55702	1	1		63.06413	2.99
77	0.4	1	75	10	26.12861	131	80	8.7	103.2	158	94	36	0	0	0	0	0		0	0	0	1468	1512	0	0				2420		0	1	0	66.27013	0.69
78	0.7	1	57	9	25.27878	152	88	7.2	119.4	199	83	63	0	0	0	0	0	0	0	0	0	1486	1522	1	0	0	1	0	286	7.772021	0	0	0	55.32495	1.04
79	0.5	1	78	19	21.33821	124	70	7	82.8	155	81	56	1	1	0	0	0	0	0	1	0	2168	2209	0	0	1	0	0	191	7.845541	1	1	0	49.56638	0.91
80	0.8	0	66	17	19.2974	168	88	8	105.4	163	83	41	1	1	0	0	0	1	1	0	0	2043	2124	1	1	1	0	0	89	6.238628	0	0		76.72289	0.48
81	0.5	0	74	16	21.45727	120	72	7.3	95.6	168	112	50	1	1	0	0	0		0	0	0	1804	1793	0	0	0	1	0			0	0	0	95.21417	0.41
82	0.6	0	69	26	23.8728	162	75	7.8	98.8	224	396	46	0	0	0	0	0	0				2133	2105	1	1	0	1	0	961	12.53591	0	0		71.74968	0.56
83	0.5	1	63	24	20.34024	126	56	8.4	101.4	193	178	56	0	0	0	0	0	1	1	1	0	1605	1831	1	1	0	0	1	1570	11.29666	1	1	0	100.1018	0.68
84	0.5	0	63	18	22.36744	118	76	10.8	107.6	207	152	69	1	0	1	0	0	1	1	0	0	1809	1817	0	0	0	1	0	509	18	0	0	0	120.6736	0.39
85	0.8	1	62	20	24.67702	122	78	7.7	176.4	233	118	33	1	1	0	0	0		1	0	0	1783	1806	0	0	0	1	0	372	9.547383	0	0		61.92499	0.98
86	0.5	0	52		22.83288	130	74	7.3	160.8	219	121	34	1	1	0	1	1	0	1	0	0	1509	1439	0	0	0	0	1						57.31138	0.72
87	0.5	1	72	14	15.625	144	62	7.4	76.2	155	74	64	0	0	0	0	0	0	0	0	0	2884	1442	1	0	0	1	0	397	7.391763	0	1	0	74.60979	0.87
88	0.5	1	61	23	25.29938	134	82	7.2	93.6	188	167	61	1	1	0	0	0		1	0	0	2046	2097	1	1	0	0	1	324	10.69837	0	0	0	76.1069	0.98
89	0.5	1	47	2	26.02758	126	74	5.8	63.8	182	396	39	1	1	0	0	0	0	0	0	0			0	0	1	0	0	363	8.661835	0	0	0	69.56476	0.86
90	0.4	1	61	6	24.91588	136	66	7.3	124.8	197	186	35	1	1	0	1	0	1	1	0	0	2557	2399	1	1	1	0	0	5160	9.739827	0	0	0	54.05831	2.85
91	0.5	1	69		23.63281	122	56	8.9	88.8	163	136	47	1	1	0	0	0	1	1	1	1	1254		1	1	0	0	1	3800	25.66855	1	1	0	53.55573	2.94
92	0.5	1	79					6.6	85.6	169	67	70	0	0	0	0	0	0	1	0	0	1885	2110		0	0	0	1	864	11.63838	0	0	0	58.55354	0.92
93	0.9	1	66	4	25.94083	128	72	7.8		178	114		1	0	1	1	1	0	1	1	1			1	1	0	1	0			0	1	0	59.59246	1
94	0.7	1	62	15	22.29081	122	76	7.2	104.6	234	342	61	1	1	0	0	0	0	0	0	0	1883	1813	1	0	0	1	0	1420	15.50894	0	1	0	70.84905	1.06
95	0.8	0	55	15	37.10575	120	68	9.4	104.4	176	103	51	0	0	0	0	0	0	0	0	0			0	0	0	1	0	5710	8.362333	0	0	0	76.57475	0.66
96	1	1	66	3	22.17096	126	62	7	157.2	228	129	45	0	0	0	0	0		1	1	1	1489	1656	1	1	0	1	0			0	0	0	69.03787	0.99
97	0.6	0	44	2	22.51644	136	60	6.7	125.6	209	147	54	0	0	0	0	0		0	0	0			0	0	0	1	0	80		0	0	0	110.5351	0.45
98	0.4	1	35	3	25.05931	142	68	7.6	180.8	314	446	44	0	0	0	1	1	0	0	0	0	1304	1252	1	0	0	1	0	1390	7.017544	0	1		108.3748	0.84
99	0.4	0	68	16	26.84703	156	70	7.3	119.4	187	78	52	1	1	0	1	0	1	1	0	1	2335	2264	1	0	0	1	0	3540	14.26599	0	0		81.46035	0.61
100	0.5	1	64	12	22.20408	134	66	8.4	133.8	216	166	49	1	0	1	0	0	1	0	0	0	2007	2056	0	0	0	0	1	1930	12.30672	0	0	0	53.51598	1.19
101	1.1	1	74	7	23.03005	140	80	6.8	98	169	110	49	0	0	0	0	0	0	0	0	0	2278	2352	1	1	0	1	0	910	9.009809	0	0	0	85.68295	1.2
102	0.4	0	65	11	25.95556	138	60	7.9	125.2	197	179	36	0	0	0	1	1	0	1	0	0	2153	2229	1	1	0	1	0	25800		0	1	0	53.78157	0.92
103	1.2	0	54	2	35.01278			6	140	215	130	49	0	0	0	0	0	0	0	0	0	1757	1787		0	1	0	0	714	8.999074	0	0		78.3607	0.67
104	0.7	1	72			130	60	8.1	96.2	166	64	57	0	0	0	0	0		0	0	1	1528	1696	1	0	0	0	1	584	13.08477	0	0	0	85.31913	0.67
105	0.7	1	67			142	62	6.3	65	156	240	43	1	1	0	1	1	0	1	0	0	2811	2732	1	1	0	0	1	1320	9.956677	1	1		69.33204	1.32
106	1	1	55	25	26.12245	136	74	7.7	138.2	206	114	45	1	1	0	0	0	1	0	0	0	1512	1498	0	0	0	1	0	274	9.950903	0	1	0	84.09272	0.82
107	0.9	1	69		20.9042	118	80	5.7		180	67		0	0	0	0	0		0	0	0	1538	1567	0	0	0	1	0	221	9.61916	0	0		73.4635	1.22
108	0.8	1	59	5	29.72108	146	98	9	197	274	150	47	0	0	0	0	0	1	0	0	0			1	1	0	1	0	1910	11.81155	0	0	0	64.72857	1.02

109	1.2	1	60	11	26.48963	130	87	7.3	118	204	40	78	1	0	1	0	0	0	1	0	0	1608	1469	1	0	0	1	0	270	8.307901	0	1		79.73545	0.87
110	0.8	0	55	11	23.80309	144	86	8.8	152.8	242	191	51	1	0	1	0	0	0	1	1	0	1522	1515	1	0	0	1	0	496	9.640666	0	0	0	82.37053	0.74
111	1	0	65		24.25332	136	72	7.2	103.8	184	96	61	0	0	0	0	0	0	1	0	1	2746	2746	1	1	0	0	1	1450	17.51825	0	1	0	103.6396	0.63
112	0.6	1	40	2	31.92794	136	76	8.6	99.8	247	526	42	0	0	0	1	1	0	0	0	0	1443	1491	0	0				3050	9.017133	0	1	0	83.0068	1.16
113	0.5	0	70	14	24.32657	124	80	9.5	142	228	155	55	0	0	0	0	0	0	1	0	0	2149	2250	1	1	0	0	1	701	22.34445	0	0	0	103.1465	0.52
114	0.6	1	77	15	19.8791	132	72	7.9	109.8	168	91	40	1	0	1	0	0	1	1	0	0	1686	1679	0	0	0	1	0	413	7.912398	0	0		69.28936	1.28
115	0.8	0	67	13	22.10429	122	85	7	135	215	115	57	0	0	0	0	0		0	0	0	1787	1844	0	0	0	1	0	2530	9.663097	0	0	0	72.35794	0.75
116	0.9	1	59		23.52234	123	77	5.2		185	154		0	0	0	0	0	0	1	0	0	1512	1488	1	1	0	1	0	272	13.34863				53.70118	1.03
117	0.4	0	64	19	23.8054	126	74	6.7	128.4	196	43	59	0	0	0	0	0	0	1	0	0	1622	1681	1	1	0	1	0	6070	14.15766	0	0	0	48.33879	0.92
118	0.6	0	25	13	21.64127	106	64	8.2	101.6	190	37	81	0	0	0	0	0	0	1	0	0	1529	1620	0	0	0	0	1	238	8.064516	0	0	0	109.2461	0.54
119	0.5	0	76	14	26.84067	130	74	7.2	89.4	166	118	53	0	0	0	0	0	0	0	0	0	2301	2326	1	0	0	1	0	888	10.58773	0	0	0	84.59506	0.6
120	0.4	1	58	11	20.81165	136	86	7.2	127.6	233	247	56	0	0	0	1	1	1	1	0	0	1744	1749	0	0	0	1	0	190	10.06899	1	1	0	60.49199	1
121	0.6	1	55	12	20.70082	132	84	7.4	251.2	133	356	41	1	1	0	0	0	0	0	0	0	1722	1863	0	0	0	1	0	135	22.32362				101.8214	0.59
122	0.7	1	63			166	86	7.6	98.2	174	179	40	0	0	0	0	0	1	1	0	1	1890	1924	1	1	0	1	0	209	12.99666	0	0		97.92925	0.95
123	0.4	0	46	20	19.47715	106	78	7	92.8	202	51	99	0	0	0	0	0	0	0	0	0	1037	1272	0	0	0	0	1	84	10.32691	1	1	0	79.70684	0.61
124	0.6	0	72	14	22.77319	122	76	7.4	64.6	163	287	41	0	0	0	0	0		0	0	0	1668	1862	1	0	0	1	0	445		0	0	0	62.0661	0.64
125	0.9	0	77	9	22.21297	116	56	8.8	136.6	197	97	41	0	0	0	0	0	1	1	0	0	2157	2225	0	0	0	1	0	469	7.522446	0	0	0	32.00501	2.19
126	0.9	1	67	18	23.73866	134	86	8.5	251.2	191	144	41	0	0	0	0	0	1	1	0	0	1441	1247	0	0	0	1	0	461	16.66056	0	1	0	65.93038	0.99
127	0.5	0	58	4	27.91724	136	78	10.8	145.6	258	247	63	0	0	0	0	0	1	0	0	0	2088	2230	1	1	0	1	0	6590	14.73585	0	0	0	89.53329	0.58
128	0.8	1	61	8	19.4674	140	66	5.8	114.6	197	52	72	1	1	0	0	0	0	1	0	0			0	0	1	0	0	255	11.62931	0	0	0	65.31729	0.74
129	0.4	0	62	1	24.56033	114	68	6.8			169	60	0	0	0	0	0	0	0	0	0	1642	1759	0	0	1	0	0	667	12.21127	0	0	0	79.58652	0.65
130	0.9	1	60	20	23.30668	133	81		118.4	222	93	85	1	0	1	0	0	0	0	0	0	1483	1411	0	0	0	1	0	214	14.64244	0	0	0	81.29583	0.77
131	0.6	0	76	12	20.96436	139	84	6.5	134.2	227	179	57	1	0	1	0	0	0	1	0	0	2122	2090	1	1	0	1	0	1070	18.92473	0	0	0	83.16599	0.6
132	0.5	1	60	3	22.95909	128	78	6.7	172.4	242	123	45	0	0	0	0	0	0	0	0	1	1545	1406	1	0	0	0	1	1390	6.695587				60.56858	3.57
133	0.5	0	74		21.45727	131	65	7.4	75.6	147	107	50	1	1	0	0	0	0	1	1	0	1096	1353	1	1	0	0	1	688	22.07308	0	0	0	62.55698	0.6
134	0.8	1	65		23.07342			6.9	109.4	190	118	57	0	0	0	0	0	0	0	0	0	2275	2295	1	1				257	8.024072	0	1		110.4022	0.62
135	0.6	0	86		21.78649	140	86	8.6		186	171		1	1	0	0	0	1	1	0	1	2347	2295	0		0	1	0	1350	34.2943	0	0		60.8808	0.7
136	0.5	1	64			156	73	6.5	160.6	234	152	43	0	0	0	1	1	0	1	0	0	1936	2021	1	1	0	1	0	7620	7.921999	1	1	0	58.17019	2.07
137	0.4	1	61	16	17.85652	142	80	7.2	76.6	123	52	36	1	1	0	1	1	0	1	0	0	1853	1938	1	1	0	0	1			0	1		82.12048	0.9
138	0.8	1	78	9	28.89549	118	80	6.6	105.6	173	157	36	0	0	0	1	0	0	1	0	0	1803	1804	0	0	1	0	0	183	13.73505	0	1		72.12985	0.73
139	0.8	0	57	21	21.00073	128	72	6.5	124	211	35	80	1	0	1	1	1	0	1	0	0	1523	1521	1	1	0	0	1	243	14.05334	0	0	0	64.70458	0.71
140	0.5	1	71	19	23.597	122	62	8.4	95.4	187	168	58	1	0	1	1	1	0	1	0	0	1997	1577	1	1	0	0	1	1200	10.56848	0	0		47.69059	3.08
141	0.5	0	51	6	28.1128	124	82	10.5	111	187	85	59	0	0	0	0	0	0	0	0	0	1340	1360	1	1	0	1	0	833	14.62317	0	0	0	91.38846	0.59
142	1.2	0	54	7	20.77562	116	74	7	126.8	200	131	47	0	0	0	0	0	0	0	0	0	1323	1343	0	0	0	1	0	564	31.95739	0	0	0	57.64156	0.66
143	0.4	0	63		17.85714	131	58	7.5		159	86		0	0	0	0	0	0		0	0	2032	2062	0	0	0	0	1	2370	14.17183	0	0		74.9695	0.53
144	0.6	0	63	10	22.317	140	78	5.9	138	218	60	68	1	1	0	0	0	1	0	0	0	1379	1462	0	0	1	0	0	369	15.2314	1	1	0	112.7719	0.44
145	0.6	1	74	4	26.46966	120	52	6	17	177	831	27	0	0	0	0	0		1	0	1	1586	1600	1	1	0	0	1	3050		0	1		23.80794	2.07
146	0.8	1	70		23.01983	130	76	9.7	95.8	186	201	50	1	1	0	0	0		0	0	1	2842	2295	0	0	0	0	1	324		0	0		115.8032	0.59
147	0.7	1	74	12	19.60519	138	58	6.3	60.2	168	54	97	0	0	0	0	0		0	0	0			0	0	0	1	0			0	1	0	70.08416	0.8
148	0.4	1	72	10	18.946	154	72	7.8	139.8	230	286	33	0	0	0	0	0	1	1	0	0	1783	1802	1	1	0	0	1	520	8.802691	0	1	0	63.83024	0.91
149	0.7	0	48	13	25.88757	136	84	7.9	133.2	218	129	59	0	0	0	0	0	1	0	0	0	1516	1526	0	0				791	7.580855	0	0	0	78.69829	0.65
150	0.6	0	67	10	21.875	142	70	9.7	126	213	110	65	0	0	0	0	0	1	0	0	0	2042	2087	1	0	0	1	0	251	28.80031	0	0		110.797	0.87
151	0.7	0	66		24.54844	146	82	6.8	108.4	189	63	68	0	0	0	0	0		0	0	0	2262	2198	1	0	1	0	0	27700	13.63774	0	0		105.7547	0.48
152	0.6	0	42					10.5	92.8	180	116	64	1	0	1	0	0		0	0	0	1284	1267		0				1250	14.72698	0	0	0	90.7082	0.58
153	0.7	1	74	36	27.70083	132	82	8.2	71.8	154	126	57	0	0	0	0	0	0	1	0	0	1626	1752	1	0	0	0	1	964	7.274676	0	1	0	66.61846	1.63
154	0.7	0	73	7		120	68	6.7		169	90		0	0	0	0	0		0	0	0	1536	1485	0	0	1	0	0			0	0	0	68.18858	0.62
155	0.6	1	84	20	19.23356	142	76	7.7	86	177	60	79	0	0	0	0	0	0	0	1	0			1	0	0	1	0		26.66899				68.49375	0.65
156	0.6	0	54	10	28.30385	108	70	9	110.4	183	148	43	1	0	1	0	0	0	1	0	0	1257	1127	0	0	0	1	0	1460	12.71841	0	0	0	104.2255	0.4
157	0.3	0	55	11	18.66201	114	60	9.2	90.8	158	146	38	1	0	1	0	0	0	0	0	0	1365	1394	0	0	0	0	1	396	13.9765	0	0	0	101.3174	0.57
158	0.4	0	62		22.98743	142	62	7	126.8	203	126	51	0	0	0	0	0	0	0	0	0	1732	1783	1	0	0	1	0	388	19.43949	0	0	0	93.6174	0.47
159	0.8	1	46	12	24.36889			12.2	128.4	190	138	34	1	1	0	0	0		0	0	0	1742	1747		0						1	1	0	94.73194	0.74
160	0.9	0	69	9	17.51463	152	102	6.1	131	209	80	62	0	0	0	0	0	0	0	0	0	1690	1704	1	1	1	0	0	383	13.78446	0	0	0	99.45793	0.5
161	0.6	0	53	9	32.65619	151	81	11.9	120.8	200	116	56	1	1	0	0	0	0	1	0	0	1584	1630	1	1	0	1	0	1130	8.02139	0	0		61.85847	0.84
162	0.6	0	54	17	22.18935	134	84	6.9	97.2	182	189	47	0	0	0	0	0	0	0	0	0	1860	1900	1	1	0	1	0	601	21.36752	0	0	0	90.43955	0.62
163	0.5	0	68	15	19.39619	120	58	7.3	168	244	40	68	0	0	0	0	0	0	0	0	0	1885	2023	0	0	0	1	0	107	4.804083	0	0	0	68.42092	0.71
164	0.7	1	75	14	24.38653	146	80	9.4	171.6	267	212	53	0	0	0	0	0	1	1	1	0	2242	2248	1	1	0	1	0	1090	16.01373	0	0	0	76.97366	0.73
165	0.7	0	56	15	25.80645	126	84	8.4	122.6	201	62	66	0	0	0	0	0		0	0	0	1336	1371	0	0	0	1	0	144	11.32353	0	1	0	94.32665	0.52
166	0.7	0	62	9	29.13632	140	86	7.4	102	159	70	43	0	0	0	1	1	0	0	0	0	1831	1947	1	1	0	1	0	429	9.917665			1	49.14817	1.06
167	0.5	1	81		21.23057	141	81	6.9		174	112		0	0	0	0	0			0	0	1930	1974	1	0	0	1	0	1370	10.9499	0	1		63.21243	1.15
168	0.6	1	69	8		143	90	6.9		154	72		0	0	0	0	0			0	0	2078	1988	1	0	0	1	0	172	19.29698				71.50554	0.79
169	0.7	1	65	10	23.50781	130	78	8.1	91.8	187	276	40	1	0	1	0	0	0	0	0	0	1457	1613	0	0	0	1	0	542	8.759022	1	1	0	60.52953	1.15
170	0.7	0	65	7		136	70	6.4	119.2	187	54	57	1	0	1	0	0		0	0	0	1668	1778	1	1						0	0	0	99.45793	0.42
171	0.5	0	64	20	28.84495	152	90	9	103	196	210	51	1	0	1	0	0	1	1	0	0	1881	1798	1	1	0	0	1	2890	12.97326	0	0		63.3615	1
172	0.5	1	46			156	96	8.2		188	200		0	0	0	0	0	1		0	0	1461	1446	1	1	0	1	0	1360	8.492315				65.36802	1.03
173	0.6	1	75	10	21.71875	130	70	7.2	104.8	154	86	32	0	0	0	0	0		1	1	0	1962	1899	1	1	0	1	0			0	1		65.43726	0.96
174	0.6	1	63	8	21.64127	122	70	7.2	122	214	95	73	0	0	0	0	0	0	1	0	0	1902	1661	0	0	0	0	1	517	8.823529	0	1	0	67.10553	0.84
175	0.5	1	70	13	29.5858	136	78	9.4	100.6	191	212	48	1	0	1	0	0	0	1	0	0	2448	2376	1	1	0	1	0	575	12.09616	1	1	0	67.02209	0.89
176	0.9	1	66	26	24.04844	132	70	12.9	113.4	225	368	38	1	1	0	0	0	1	1	1	0	2042	2005	1	1	0	0	1	436	16.19365	0	1	0	84.78441	0.85
177	0.4	0	53	11	33.61095	122	80	14	168.2	255	159	55	1	0	1	0	0	0	0	0	0	1433	1547	0	0	0	1	0	478	11.89181	1	1	0	68.85753	0.77
178	1	0	57					5.3	108.8	224	356	44	1	1	0	1	1	0	0	1	0	1775	1834		0				2210	13.28168	0	0	0	43.49769	1.07
179	0.7	1	75	16	20.70313	110	60	9	114.2	199	64	72	0	0	0	0	0	0	0	0	0	1886	1963	0	0	0	1	0	227	15.76065	1	1	0	87.53657	0.54
180	0.7	0	59	11	25.96454	126	66	11.9	136.8	233	246	47	1	0	1	0	0	0	1	0	0	1568	1562	0	0	0	1	0	499	7.874016	1	1	0	75.04731	0.81
181	0.8	1	70	14	27.96802	124	76	6.8	122.4	180	103	37	0	0	0	0	0	0	1	0	0	1621	1598	0	0	0	0	1	3840	12.33056	0	1	0	49.65977	1.27
182	0.8	0	59	2	26.22268	126	86	6.2	99	185	145	57	0	0	0	0	0		0	0	0	1362	1379	1	0	1	0	0					0	76.02666	0.55
183	0.5	0	37	5	24.06405	146	80	6.9	89.2	165	169	42	0	0	0	0	0		0	0	0			1	0	0	1	0	14300		0	1		110.9936	0.47
184	0.5	1	53			160	100	10.8	103.6	196	277	37				1	1	0	1	1	0			1	0	0	1	0			1	1	1	65.66061	7.4
185	1.1	1	81	7	20.54569	136	80	5.9	122.8	208	76	70	0	0	0	0	0	0	0	0	0	2006	2162	0	0	1	0	0	84	7.439077	0	1	0	75.29212	0.79
186	0.6	0	53	8	23.82813	120	76	7.3	101.4	179	68	64	0	0	0	0	0	0	0	0	0	1260	1166	0	0	0	1	0	119	9.527934	0	0	0	86.509	0.42
187	0.3	0	53	7	26.47918	144	68	6	136	213	115	54	1	1	0	1	1	0	1	0	0	1343	1350	1	0	0	1	0	127	8.45666	0	0	0	69.95427	0.98
188	0.4	0	63	8	24.15766	154	84	6.8	93	174	85	64	1	0	1	0	0	1	1	0	0	1911	1921	1	0	0	1	0	128	10.10101	0	0	0	73.97521	0.58
189	0.7	0	59			125	75	8	146.4	233	128	61	0	0	0	0	0	0	0	0	0	1338	1374	0	0	0	1	0	115	17.76981	0	0	0	83.88686	0.63
190	0.6	0	64	20	24.71434	112	70	5.9	84.4	139	43	46	0	0	0	0	0	0	0	0	0	1638	1759	1	1	0	1	0	155	13.0246	0	0		66.75922	0.54
191	0.6	1	64		20.76665			6	131.4	254	193	84	0	0	0	0	0		1	0	0	2780	2957		0				116		0	1	0	66.80291	1.23
192	0.8	1	84	3	25.20398	116	66	7.3	95.2	164	64	56	0	0	0	0	0	0	1	0	0	2167	2065	0	0	0	1	0	772	8.724231	0	1		58.20117	0.85
193	0.5	1	67		20.02884			8.6	58.6	169	132	84	0	0	0	0	0			0	0	2093	2175			0	1	0			0	0	0	79.67445	0.75
194	0.7	1	40	1	23.1274	122	52	6.5	124.4	188	68	50	0	0	0	0	0		0	0	0	1080	1069	0	0	1	0	0	701	8.101598	0	0		69.57965	0.92
195	0.6	1	68	3	25.59374	120	80	4.9	69.4	128	73	44	0	0	0	1	0	1	0	0	0	1889	1726	0	0	0	1	0	312	7.629428	0	1	0	23.80039	2.16
196	0.8	1	47	4	22.06416	126	60	7.1	251.2	183	208	41	1	0	1	0	0		0	1	0	1395	1400	1	1	0	0	1			0	1		85.72458	0.64
197	0.8	0	55	5	23.40192	130	89	9.9	136.2	241	74	90	0	0	0	0	0	0	0	0	0	1544	1664	0	0	0	1	0	868	10.54945	0	0	0	99.05755	0.49
198	0.3	1	45	16	22.26813	120	68	8	72.6	224	582	35	1	1	0	0	0	0	1	0	0	1319	1415	1	1	0	1	0	939	8.548748	1	1		64.35794	0.89
199	0.5	1	76	55	24.39024	114	62						0	0	0	0	0	1	0	0	1	2242	2256	1	0	0	0	1	58100	330.041	0	0		70.48969	1.02
200	0.6	0	70	3	29.89969	116	78	6	144.2	225	129	55	0	0	0	0	0		0	0	0	1484	1630	0	0	1	0	0			0	0	0	66.73013	0.49
201	0.5	0	54	5	22.35434	136	76	5.6	150.6	247	152	66	1	1	0	1	1	0	1	0	0	2505	2313	1	0	0	0	1	286	8.850066	0	0	0	40.30924	1.89
202	1	1	57	6	28.93407	134	92	7.3	48	109	20	57	1	1	0	0	0	0	1	0	0	1756	1783	1	1	1	0	0	161	10.99493	0	1	0	95.78215	0.56
203	0.5	1	67	6	22.03173	126	60	6.7	122	183	75	46	0	0	0	0	0	0	0	0	0			0	0	0	1	0	50	13.61753	0	0		44.68451	1.45
204	1.1	1	62			125	82	6.3	107.2	247	259	88	0	0	0	0	0		0	0	0	1417	1428	0	0	1	0	0			0	1	0	96.69954	0.75
205	0.4	1	64	5	29.41176	140	78	6.5	78	150	115	49	1	0	1	1	1	0	1	0	0			0	0	0	1	0	348	6.685914	0	0	0	60.79947	1.12
206	0.5	0	33	7	19.56296	88	54	9.2	78.2	159	64	68	0	0	0	0	0	0	0	0	0	1121	1153	0	0				608	14.34017	0	0	0	95.37673	0.52
207	0.4	1	54	5	21.95248	114	68	9.8	126.8	204	171	43	0	0	0	0	0	0	0	0	0	1431	1405	0	0				641	7.414382	0	1	0	99.56596	0.72
208	0.8	1	62	12	23.03005	124	80	7.9	130	190	80	44	0	0	0	0	0	0	0	0	0	1492	1315	0	0	0	1	0	96	6.129032	0	1	0	88.25253	0.68
209	0.6	0	68					7.1	103.8	188	96	65	0	0	0	0	0	0	0	0	0	1663	1686		0				203	15.07862	0	0		67.2876	0.7
210	0.7	1	65	6	25.0995	165	94	6.5	118.4	173	48	45	1	0	1	0	0		1	1	0	1724	1673	1	1	0	1	0	1490	10.96957	0	1		69.04851	0.79
211	0.4	1	79	4	19.72387	148	76	5.5	97	165	45	59	0	0	0	0	0		0	0	0			1	1	0	1	0	362	12.99134	0	1	0	57.8866	1
212	0.5	0	54	7	30.26183	126	62	11.9	168.2	259	189	53	1	1	0	1	1	0	1	0	1	2007	2029	1	1	0	1	0	2990	13.78346	0	0		46.13433	3.46
213	0.6	1	68	6	28.3737	114	80	7.7		164	107		1	1	0	1	1	0	1	1	0	1812	1487	0	0	1	0	0	81	10.85814	1	1	0	53.44489	1.06
214	0.4	1	55	8	19.43697	114	73	8.5	108.8	214	71	91	0	0	0	0	0	0	0	0	0	1401	1549	0	0	0	0	1	93	14.2798	1	1		84.13963	0.79
215	0.5	0	76		29.55255	173	85	6.6		224	157		0	0	0	0	0			0	0	1820	1728	1	0	1	0	0	1780	11.97322	0	0		43.92118	1.07
216	0.3	1	61		26.59656	152	74	8.8	93.8	211	396	38	1	0	1	1	1	0	1	0	1	1711	1745	1	1	0	1	0	5210	18.79094	1	1	0	21.12402	2.64
217	0.8	0	40	5	33.94931	120	88	8.5	199.4	284	193	46	0	0	0	0	0	0	0	0	0	1169	1159	0	0	0	1	0	881	11.20317	0	0	0	103.8899	0.46

218	0.5	1	72	10	27.29322	142	78	6.9	83.8	148	131	38	1	1	0	0	0	1	1	0	1	1638	1611	1	0	0	0	1	183	5.638088	0	0	0	75.67047	0.56
219	0.8	1	60	9	22.85289	195	100	7.2	187.6	258	147	41				1	1	1	1	0	0	2117	2074	1	0	0	1	0	150	7.385094	0	1	0	69.23141	1.09
220	0.8	0	69	6	22.18935	126	62	7.4	102	168	95	47	0	0	0	0	0		1	0	0	1673	1913	0	0	0	1	0	302	11.65501				56.60772	0.82
221	1.1	1	74		19.57168					218	108		1	1	0	0	0	1	0	1	0	2004	2034	1	1	1	0	0	728	10.38682	0	1	0	60.35738	1.08
222	0.8	1	63					9	93.2	188	214	52	0	0	0	0	0	1	1	0	0				0	0	1	0	1020	11.06057				78.62672	0.72
223	1	1	34	2	23.88946	126	72	5.4	68.8	144	96	56	0	0	0	0	0		0	0	0	1176	1327	0	0	0	0	1	174	13.39309	1	1	0	109.2802	0.64
224	0.3	0	68	20	24.32323	130	75	5.9	132.6	211	202	38	1	1	0	1	1	0	1	0	0	1645	1797	1	1	0	0	1	687	14.54545	0	0	1	22.72776	5.62
225	0.6	1	36			122	70	5.3	79.6	159	62	67	1	0	1	0	0		1	0	0			0	0	1	0	0	177	15.14496	0	0	0	91.77042	0.82
226	0.5	1	71	5	22.67995	124	78	6.9	127.6	215	167	54	0	0	0	0	0	0	0	0	0	2234	2074	0	0	0	1	0	348	6.900945	0	0	0	79.58973	0.7
227	0.7	1	64	4	21.48438	142	76	6.6	94.8	183	86	71	0	0	0	0	0		0	0	0			1	0	0	1	0			0	1	0	108.768	0.63
228	0.6	1	50	12	21.96712	136	72	6.8	146.8	231	211	42	0	0	0	0	0	1	0	0	0	1609	1668	1	1	0	1	0	421	9.542356	1	1	0	116.7536	0.62
229	0.5	0	53	20	28.55851	126	76	12.6	97.4	191	248	44	1	1	0	0	0	1	1	0	0	1510	1506	0	0	0	0	1	671	6.820119	0	0	0	104.7862	0.5
230	0.7	1	65	40	26.92744	142	76	7.5	176	261	155	54								0	0	1633	1598	1	0	1	0	0			0	0	0	54.78865	1.18
231	0.4	0	63			120	52	5.9	168.2	239	94	52	0	0	0	0	0	0	1	0	0			1	0	0	1	0	2800	16.63042	0	0	0	78.11198	0.71
232	0.5	1	57	2	22.86585	104	60	5.4		180	453		0	0	0	0	0		0	0	0	2297	2266	1	0	1	0	0	3460	14.56186	0	1	0	80.91794	0.65
233	0.6	1	54	4	25.35154	136	84	6.9	149.4	224	113	52	0	0	0	0	0	1	0	0	0	1676	1739	0	0	0	1	0	1050	4.958246	0	1	0	73.57057	0.81
234	0.5	0	60	4	22.60715	110	76	6.7		212	246		0	0	0	0	0		0	0	0	1383	1436	0	0	1	0	0			0	0	0	89.27457	0.59
235	0.9	1	59	23	25.71166	110	70	7.8	124.2	217	264	40	1	1	0	1	0	1	1	0	0	1777	1785	1	0	0	0	1	6500	42.33766	0	1	0	45.29994	1.1
236	0.5	0	43	2	26.10656	113	70	5.9	64.8	166	356	30	0	0	0	0	0		0	0	0	1328	1376	0	0	1	0	0			0	0	0	77.02771	0.65
237	0.5	1	66		23.24219	136	72	7.1	109.2	174	89	47	0	0	0	0	0		0	1	0			1	1	0	1	0			0	0		73.40236	0.68
238	0.7	0	64		23.32752			7.2	92.8	192	116	76	1	1	0	0	0	1	1	0	0	2151	2290	1	1	0	1	0	469	15.05531				78.86463	0.64
239	0.8	1	57		24.15882					249	440		1	0	1	0	0	1	0	0	0	1701	1785	1	1				698	10.0525	1	1	0	53.18606	1.29
240	0.6	1	74	8	26.34649	140	68	6.4	96.8	174	151	47	0	0	0	1	1	0	0	0	0	1661	1729	1	1	0	1	0	1290	13.0593	1	1	0	75.07777	1.69
241	0.9	0	59	23	20.31592	144	62	7.3	101.8	191	51	79	1	0	1	0	0	0	1	0	0	1684	1604	1	0	0	0	1	3250	9.128065	0	0	0	86.4605	0.52
242	0.6	0	41	1	29.47584	136	66	5.9	130.2	216	194	47	0	0	0	0	0		0	0	0	1375	1418	0	0	1	0	0	433	19.87179				97.59244	0.48
243	0.4	0	59	6	14.04115	156	86	9	75.4	151	58	64	1	1	0	1	1	0	1	0	0	2170	2252	1	1	0	0	1	47600	17.04731	0	0		97.89305	0.66
244	0.5	1	64	13	28.66414	124	75	8.3	101.2	204	324	38	1	1	0	1	1	0	1	0	0	1863	1818	1	1	0	0	1	1380	6.504904	0	1	0	73.08625	1.21
245	0.5	0	81	14	22.35556	165	80	5.4	156.6	222	57	54	1	1	0	0	0		0	0	0	2147	2089	1	0	0	0	1			0	0		65.49635	0.48
246	0.9	1	41	13	26.354	126	82	7.5	77	172	205	54	0	0	0	0	0		0	0	0	1388	1422	0	0	0	1	0	1400	12.84158	0	0	0	87.6963	0.91
247	0.5	0	74	40	16.88872	136	60	12.3	70	201	425	46	1	1	0	1	1	0	1	0	1	2406	2623	0	0	0	1	0	775	28.42427	0	0		80.86946	0.59
248	0.8	0	60	11	26.14269	124	62	10.1	88.8	163	126	49	0	0	0	0	0	0	1	1	0	1708	1693	1	1	0	0	1	1050	18.97233	0	1	0	62.82146	1.26
249	0.9	0	43	8	14.93889	114	63	11.6	137.8	210	96	53	0	0	0	0	0		0	0	0	1189	1253	0	0	0	0	1			1	1		104.93	0.49
250	0.4	0	56	19	25.91513	173	100	11.6	149.6	227	187	40	1	1		1	1	0	1	0	0	1664	1745	1	0	0	0	1			0	0	1	71.9752	2.82
251	1.1	0	31		18.46606			8.2	94.4	184	63	77	0	0	0	0	0		1	1	0	1085	1151	1	1						0	0		82.09764	0.64
252	0.6	0	68	18	24.41331	161	85	10.4	125.4	198	153	42	0	0	0	0	0		0	0	1	2228	2004	1	1	0	1	0			0	0		63.09266	1.26
253	0.7	1	60	2	23.28125	126	74	6.6	75	143	85	51	1	1	0	0	0		1	1	0	1450	1464	1	1	0	1	0			0	1	0	54.00584	1.06
254	0.3	1	43	3	25.68956	170	97	11.8	151.4	350	788	41	1	1	0	1	1	0	1	0	1	1718	1656	1	1	0	1	0	36700	7.507012	0	1	1	61.84601	13.48
255	0.5	1	53	2	27.39092	136	72	7.2	129.4	229	248	50	0	0	0	0	0		0	0	0	1670	1578	1	0	0	1	0						90.2938	0.67
256	0.5	1	61	10	24.24242	106	76	7	121.8	214	246	43	0	0	0	0	0	0	0	0	0	1350	1259	0	0	0	1	0	815	8.572705	1	1		81.29583	1
257	0.7	0	70	12	20.50493	128	78	7.3	157.8	235	61	65	0	0	0	0	0	0	0	0	0	1955	1924	0	0	0	1	0	104	12.57239	0	0	0	86.61545	0.52
258	0.7	1	31	8	17.05618	106	54	7.4	86.6	142	67	42	0	0	0	0	0	0	1	0	0	1423	1433	0	0	0	1	0	235	15.46823	0	0	0	101.8747	0.77
259	1	1	58		27.81321	143	82	9.3	123.6	190	122	42				0	0		1	1	0	1450	1483	1	1	1	0	0	163	8.417022	0	1		59.83707	1.13
260	0.5	0	54	2	31.44528	142	72	7.5	126.4	207	178	45	0	0	0	0	0		0	0	0			1	0	0	1	0	1760	16.25767	0	0		101.8524	0.42
261	0.5	1	55	2		136	84	6.6	114.6	226	217	68	0	0	0	0	0	0	0	0	0	1949	2331	0	0	0	0	1	6910	11.89296	1	1	0	73.3725	0.83
262	0.3	1	63	3	20.0796	126	72	7	113.6	166	87	35	0	0	0	0	0	1	0	0	0	1548	1516	0	0	0	1	0	35000	9.477009	1	1		91.55144	0.86
263	1	1	57			126	56	5.9	121.6	211	52	79	0	0	0	0	0		0	0	0			0	0	1	0	0			0	0	0	66.94034	0.96
264	1	1	64	20	19.72104	164	84	7.3	127.2	224	44	88	1	0	1	0	0		0	0	0	2251	2276	1	0	0	1	0	1170	10.41323	0	1	0	67.63384	0.94
265	1.1	1	56	3	20.95727	156	100	7.9	93.6	152	82	42	0	0	0	0	0	1	0	0	0			1	0	0	1	0		12.74036	0	1		101.2962	0.6
266	1	0	65		23.19109	132	69	7.4	141.2	211	74	55	1	0	1	0	0	1	1	0	0	1249	1289	0	0	0	1	0	406	12.165	1	1	0	68.16461	0.55
267	0.8	0	70	1	21.35276	152	82	5.3	102.6	169	67	53	0	0	0	0	0	1	0	0	0	1672	1652	1	0	1	0	0	89	11.95446	0	1	0	75.43813	0.71
268	0.7	0	52	2	22.14844	134	74	6.2	154.4	241	68	73	0	0	0	0	0		0	0	0	1439	1474	0	0	1	0	0			0	0	0	66.72712	0.65
269	0.5	1	74	18	22.4323	124	84	6.8	151	221	120	46	0	0	0	0	0		0	0	0	1785	1757	0	0	0	1	0			0	0	0	58.98309	0.83
270	0.5	0	65	15	16.74952	136	76	5.9	149.8	213	91	45	1	0	1	0	0	1	1	0	0	1837	1793	0	0	0	0	1	664	23.0595	0	0		90.37707	0.65
271	1	0	52	15	25.23634	136	66	7.2	144.4	226	108	60	0	0	0	0	0	0	1	0	0	1786	1848	0	0	0	0	1	3550	12.50657	0	0		70.33775	0.69
272	0.4	0	62	5	25.65677	128	86	9.6	91.4	188	168	63	0	0	0	0	0		0	0	0	1416	1424	0	0	0	1	0			0	0		63.79008	0.62
273	0.5	0	66	11	21.98989	130	72	5.2	155.8	235	131	53	1	1	0	0	0		0	0	0	2474	2242	1	1	0	1	0	500	14.32231	0	1	0	46.09302	0.84
274	0.6	1	60	2	18.87971	126	66	10.8	71.2	178	39	99	0	0	0	0	0	0	0	0	0	1824	1862	1	0	0	0	1	112	14.29646	0	1	0	85.8125	0.72
275	0.6	1	76	2	25.28257	128	68	5.6	73.4	135	78	46	0	0	0	0	0	1	0	1	0	2169	1867	1	1	0	1	0	787	18.68639	0	1	0	53.6258	4.22
276	0.7	1	54		19.20351	115	79	9.6	96.4	179	118	59	1	1	0	0	0	1	1	0	0	1358	1325	0	0	0	0	1	214	17.26138	1	1		114.203	0.62
277	0.8	0	59	30	21.78649	114	60	6	119	219	70	86	0	0	0	0	0	0	1	0	0			0	0	0	0	1	353	11.89446	0	0		83.88686	0.55
278	0.5	1	64	4	21.6041	112	78	6.6	133.6	208	62	62	1	1	0	0	0	0	0	0	0	1342	1348	0	0	0	1	0	244	4.834051	0	1	0	71.16501	0.76
279	0.6	0	65			134	86	7.8	136.4	185	64	61	1	1	0	0	0	0	1	0	0	2110	2215	1	0	0	1	0	97	14.38849	0	0	0	90.37707	0.49
280	0.4	0	61	21				8.5	109.4	198	193	50	1	0	1	0	0		1	0	0				0	0	0	1			1	1	0	105.0546	0.45
281	0.4	1	43					9.9	95.8	235	481	43	1	0	1	0	0		0	0	0				0						1	1		94.42229	0.7
282	0.6	1	35	14	20.76125	144	86	7	122.8	189	91	48	1	1	0	0	0	1	1	0	0			1	0	0	0	1	1280	9.402092	1	1		115.9246	0.86
283	0.3	1	57	20	26.83016	136	86	6.7	131.2	225	309	32	1	1	0	1	1	0	1	0	0	2166	2019	1	1	0	1	0	626	5.907256	0	1	1	32.75479	11.7
284	1	1	60	14	25.31545	128	72	8	133.2	203	119	46	0	0	0	0	0	1	0	0	0	1591	1703	1	0	0	0	1	4740	11.39159	0	1	0	58.62242	1.17
285	0.7	0	58		21.44474	142	82	6.3	143.6	241	152	67	1	1	0	0	0	0	1	0	0	1865	2021	1	1	1	0	0	204	7.578367	0	1	0	62.14452	0.67
286	0.6	1	61			152	76	5.8	87.8	185	361	25	1	0	1	1	1	0	1	0	0	1617	1547	1	1	0	0	1	215	6.20229	0	1	1	11.87751	10.46
287	0.8	0	57	18	26.01281	159	85	7.7	167	250	120	59	1	1	0	0	0		1	0	0	1627	1633	1	1	0	1	0	317		0	0	0	88.15995	0.53
288	0.7	0	69	11	17.84652	132	72	9.4	156	239	90	65	1	1	0	1	1	0	1	0	0			0	0	0	0	1	397	14.23488	0	0	0	99.45793	0.65
289	0.7	1	59	1	23.02041	141	90	8.1	115.4	180	108	43	1	0	1	0	0	1	1	0	0	2138	2077	1	0	0	1	0	1240	18.71028	1	1	0	113.514	0.9
290	0.6	0	56	1	19.7777	136	78	6.3	131.8	208	61	64	0	0	0	0	0	0	0	0	0			0	0	1	0	0	115	9.64991	0	0	0	69.98265	0.58
291	0.9	1	62					5.8	104.4	176	118	48	0	0	0	0	0		0	0	0				0	1	0	0	84		0	0		69.99129	0.86
292	1.1	1	55			136	72	6.9	154.6	241	217	43	1	0	1	1	1	0	1	0	0			0	0	0	1	0			0	1	1	84.13963	2.2
293	0.5	0	61	20	23.53546	138	90	8	64.2	157	139	65	1	1	0	1	1	0	1	0	0	1848	1809	1	1	0	1	0	326	14.72172	1	1	1	90.10499	3.73
294	0.6	1	46	15	21.80233	136	92	10.9	106.4	231	453	34	1	1	0	1	1	0	1	0	0	1600	1547	1	1	0	0	1			0	1	1	53.94501	11.71
295	0.6	1	70	30	23.18339	134	66	6	115.4	192	83	60	0	0	0	0	0		1	0	0			0	0	0	1	0			0	0	0	84.6684	0.75
296	0.7	0	56		18.76525	130	80	7.2	77.4	156	103	58	1	1	0	0	0		1	0	0	2243	2232	1	1	0	1	0	367	15.17451	0	0	0	101.3174	0.65
297	0.6	0	65	10	20.2848	123	53	6.2	118.6	225	62	94	0	0	0	0	0	0	0	0	0	1903	1975	0	0	1	0	0	862	9.881423				78.51449	0.62
298	0.6	0	57	4	18.13202			8	176.2	267	99	71	0	0	0	0	0		0	1	0			0	0	0	1	0	1220	17.73597	0	0	0	84.72126	0.51
